# Supplementary material for: Patient perspectives on interpersonal aspects of healthcare and patient-centeredness at primary health facilities: A mixed methods study in rural Eastern Uganda
Source: PLoS One. 2020 Jul 30;15(7):e0236524. doi: 10.1371/journal.pone.0236524 (PMC7392339; doi:10.1371/journal.pone.0236524)
Supplement: S1 Appendix — A compilation of the informed consent forms and tools used in qualitative and quantitative data collection (both in English and translated into Lusoga). (DOCX) [file pone.0236524.s001.docx]

**Supplementary File S1 Appendix.** Translated tools to measure patient perceptions of PCC in Uganda

**Tool 3h: PATIENT INTRODUCTION SHEET** *(in the waiting room before entry to the consultation room, this can also be done by the health worker before the consultation)*

*Research title***:**

Okutegeera engeri edh’endhidandaba eyetololera ku mulwaire ku mutendera gw’amalwaliro agasookerwaku mu maserengeta g’eirungu lya Sahara-Ensonga ya Uganda

*Understanding patient-centred care approaches at the level of primary health care facilities in sub-Saharan Africa: the case of Uganda*

**Enhandula**

**Bwekiba kikolebwa alikunoonereza**

Okusookera irala, webale inho olw’ebiserabyo niinze wano buti. Amainha gange nhinze anoonereza okuva mu Institute of Tropical Medicine mu Antwerp. Mu Uganda tuli kukolera ghalala ni Makerere University. Tuli kukola okunoonereza ku ngeri edhisobola okukozesebwa okukakasa nga empeereza eweebwa mu malwaliro agasookerwaku eta inho eisira ku mulwaire; era buzibu na mikisaki byetunasobola okubitamu. Nandienze okubuzaaku ebibuuzo ebyekuusa ku kino nga omaze okwebuzaaku, kino bwekiba kirungi ndidha kukulindira ku___________________(kuluya aghasembayo era nkukulemberemu mu kifo eky’ekusifu muno) nga omaze okuyaalira omusawo, tubonagane.

*Introduction*

*If done by the researcher*

*First of all, thank you very much for your time with me right now .My name is _______________a research assistant working for the Institute of Tropical Medicine in Antwerp. In Uganda we are working together with Makerere University*

*We are conducting a research study looking at approaches that can be taken to ensure the care provided at primary health care facilities is more focussed on the patient; and what challenges and opportunities would be experienced. I would like to ask you some questions in regard to this after your consultation, if this is okay with you, I will be waiting for you at _________________(outside the last point of care and guided to a private location within the facility) after your visit with the health worker, see you soon* ***.***

Bwekiba kikolebwa omusawo (okugeza nga bamaze okwogera kuby’obulamu)

Tusangaire okubonaku olwaleero ku irwaliro, bano n’abanoonereza nga balikolera aba Insititute of Tropical Medicine mu Antwerp ne eitendekero eikulu ery’e Makerere.

*If done by the health worker (for example after a health talk)*

*Welcome to the facility today, these are research assistants working for the Institute of Tropical Medicine in Antwerp and Makerere University*

Bali kukola okunoonereza ku ngeri edhisobola okukozesebwa okukakasa nga empeereza eweebwa mu malwaliro agasookerwaku eta inho eisira ku mulwaire; era buzibu na mikisaki byetunasobola okubitamu. Bandienze okubuuzaaku ebibuuzo ebyekuusa ku kino nga omaze okwebuzaaku, kino bwekiba kirungi baidha kukulindira ku___________________(kuluya webaidhandabira awasembayo era baidha kukulagirira era bakulemberemu mu kifo eky’ekusifu muno) nga omaze okukyaalira omusawo, webale olw’enkolaganayo.

*They are conducting a research study looking at approaches that can be taken to ensure the care provided at primary health care facilities is more focussed on the patient; and what challenges and opportunities would be experienced. They would like to ask you some questions in regard to this after your consultation, if this is okay with you, they will be waiting for you at _________________(outside the last point of care and guided to a private location within the facility) after your visit with the health worker, thank you for your cooperation.*

**Tool 3i: Patient Information Sheet (after receiving care)**

Okutegeera endabirira ey’abalwaire ku mutendera ogw’amalwaliro agasookerwaku mu maserengeta ga Africa: Ensonga ya Uganda

*Understanding patient-centred care approaches at the level of primary health care facilities in sub-Saharan Africa: the case of Uganda*

**Enhinonola enhangu ku kunonenereza**

Kuno n’okunoonereza okuli kulingirira ku ngeri edhisobola okukozesebwa okukakasa nga empeereza eweebwa mu malwaliro agasookerwaku eringa inho ku mulwaire; era buzibu na mikisaki byetunasobola okubitamu ku malwaliro agasookerwaku mu maserengeta ga Sahara

*Lay explanation of research title:*

*This is a study looking at approaches that can be taken to ensure the care provided at primary health care facilities is more focussed on the patient; and what challenges and opportunities would be experienced at primary health care level in a Sub-Saharan context*

Okusookera irala, webale inho olw’ebiserabyo okutyama niinze wano buti. Amainha gange nhinze__________________anoonereza okuva mu itendekero lya Makerere University

*First of all, thank you very much for your time in sitting down with me right now .My name is _______________a research assistant working with Makerere University*

Tuli kukola okunoonereza ku ngeri edhisobola okukozesebwa okukakasa nga empeereza eweebwa mu malwaliro agasookerwaku eringa inho ku mulwaire; era buzibu na mikisaki byetunasobola okubitamu. Nandienze okubuuzaaku ebibuuzo ebyekuusa ku kino.Nenda otegeere nti ghazira kituufu oba kifu mu buli ky’okoba. Era, okunoonereza kuno tikuja kumanibwa. Kino kitegeeza nti buli ky’okoba tikiidha kukunoonenkerezebwaaku. Okunoonereza kwidha kutwala kitundu kya saawa. Kinaaba kirungi singa mpandiika byonandiramu? Nga bwobona, nnina olupapula n’ekalamu okuwandiika, kino kidha kunnamba okwiidhukira. Kansubire nga tofaayo.

*We are conducting a research study looking at approaches that can be taken to ensure the care provided at primary health care facilities is more focussed on the patient; and what challenges and opportunities would be experienced. I would like to ask you some questions in regard to this. I would just like you to know that there is no right or wrong in anything you say. Also, this survey is anonymous. This means that anything you say will not be traceable back to you. The survey will take about a half an hour. Would it be okay if I wrote your answers down? As you can see, I have some paper and pencil to write, this will help me to remember. I hope you do not mind.*

Nga okaali kusalagho kwenhigira mu kunoonereza kuno, kyamugaso okusoma olupapula luno. Olina eidembe okubuuza ebibuuzo ekiseera kyonakyona. Wandienze mbitte mu lupapula luno niighe? *[if yes, Continue with this informed consent form, if no terminate the conversation here and record refusal]*

*Before you decide to participate in this study, it is important that you read this form. You have the right to ask questions at any time. Would you like me to go through the form with you? [if yes, Continue with this informed consent form, if no terminate the conversation here and record refusal]*

**Omugaso n’okwinhonola ku kunoonereza**

Kuno n’okunoonereza okuzuula emikisa n’obuzibu mu kutuusa endabirira ey’abalwaire mu malwaliro agasookerwaku mu Uganda. Twidha kuba nga twogera n’abalwaire, abakola mu by’obulamu (abasawo n’abakola egy’okuwereza abantu), abakola amateeka, abasomesa eby’obulamu era n’abanoonereza abenhigira mu malwaliro munaana mu Uganda. N’olwekyo, buli gwekigemaku aidha kwetebwa okwenhigira mu kutegeka n’okukola engeri edh’okutaasa edhigwana, emitendera egy’okulondoola gyidha kukolebwa okupima enkyuuka etereibwabwo okutaasa ku mutindo ogw’empeereza eweebwa mu bifo ebidhandabirwamu ebya ga gavumenti n’eby’obwanakyeegha.

*Purpose and description of the study*

*This is a study to explore opportunities and challenges in the delivery of patient-centered primary health care services in Uganda. We shall be talking with patients, health care workers (doctors, nurses and social workers), policy makers, health educators and researchers involved in primary level care at 8 facilities in Uganda. Consequently, all stakeholders will be invited to participate in the design and implementation of an appropriate intervention, follow-up phases will be conducted to assess the impact of interventions on the quality of versatile health care services provided at both public and private primary care facilities*

Bwoikiriza okwenhigira mukunoonereza kuno, oidha kubuuzibwa ebibuuzo kungeri gyowuliramu kukutabagana kwo n’omusawo wo, era n’obusobozi bwo okukola okusalawo okwaghalala okukuuma obulamu bwo oba okusobola obulwairebwo.

*If you accept to participate in this study, you will be asked questions on how you feel about the interaction between you and your health care provider; and your ability to make joint decisions to maintain your health or manage your illness*.

**Okutegeera okukeberwa mu kunonerera**

Bwoikiriza okwenhigira mu kunoonereza kuno, ezira kukeberebwa kwa munda kugya kukolebwa. Anoonereza aidha kubaaku n’okwogeramu niighe okutono nga okaali (nga eno bwolinda okwingira) era ng’omaze okwebuuzaku. Bwoba toyenda kwiiramu ekimu ku bibuuzo, osobola okwogera era ndidha kugya ku kibuuzo ekiiraku. Oluwayo lwa leero nga luwoire, era oidha kwetebwa mu luwayo olw’abangi n’abalwaire abandi mu kifo eky’aghalala lu lunaku n’esaawa eby’endhawulo.

*Examinations in the context of the study*

*If you accept to participate in the study, no invasive tests and examinations will be performed. The researcher will have a brief discussion with you before (while waiting to go in) and after your consultation. If you do not want to answer any of the questions, you may say so and I will move on to the next question. After today’s session, you will also be invited for a focus group discussion with other patients at a central location on a different day and time.*

**Okwenhigiramu okwekyeyendeire**

Wenhigiramu kyeyendeire mu kunoonereza kuno era olina eidembe okudhema okwenhigira mu kunoonereza kuno. Okusalawokwo okwenhigira mu kunoonereza kuno oba bbe, tikwiidha kuba na kyamaanhi kyekukola ku bwidhandabi bw’oduna ku irwaliro. Era olina eidembe okulekera okwenhigira mu kunoonereza kuno ku kiseera kyonakyona, nibwooba oikiriza.

***Voluntary participation***

*You participate entirely voluntarily in this study and you have the right to refuse to participate in the study. Your decision to participate in this study or not, will have no influence whatsoever on the care you get at this facility. You also have the right to stop you participation in the study at any time, even after you have given consent.*

**Obuzibu n’obukaluubirivu**

Tighaabe bukaluubirivu okwenhigira mu kunoonereza kuno.

*Risks and inconveniences*

*There will be no physical risks to participating in this study*.

**Ebirungi**

Titusobola kukakasa nti oidha kuganulwa mu buligho okuva mu kwenhigira mu kunoonereza kuno leero. Bwoikiriza okwenhigira mu kunoonereza kuno, amawulire okuva mu kunoonereza kuno gasobola okuyamba mu kulongosa amagezi ku kwogerezagania wagatiwo n’abalwairebo era n’okuyamba abalwaire abandi yebwiidha.

*Advantages*

*We cannot confirm that you will personally benefit directly from your participation in this study today. If you consent to participate in this study, the information resulting from this study can contribute to better knowledge on the interaction between you and your patients and help other patients in future****.***

**Okuliyirwa**

Ghazira kuliyirwa mu sente kuliwo kulw’okunoonereza kuno leero kubanganokubuuzibwa kuli kukolebwa ku irwaliro, aye osaana wenhigire mu kunoonereza okwiiraku okwaghalala; oidha kusasulwa entambula.

***Compensation***

***There is no monetary compensation available for this study today because the interview is done at the facility, but should you agree to participate in the subsequent focus group discussion; you will receive a transport reimbursement***

**Okukuuma obulamubwo obw’ekyaama**

Ebikugemaku n’okwenhigirakwo mu kunoonereza kuno biidha kutwalibwa nga bya kyaama. Toidha kumanhikibwa ku maina oba mu ngeri eyindi ey’okumanibwa mu mpapula, ebinaava oba ebinafulumizibwa nga bigema ku kunoonereza kuno. Ebikugemaku bisigala nga kyaama olw’okubanga amawulire ku muntu agidha kutebwaaku namba eyendhawulo (n’olwekyo gaidha kukwekwebwa). Amawulire agakugemaku gaidha kusengedhebwa era gekeeneeneezibwe n’ebyuuma ( mu kyuuma ki kalimagezi) oba mu ngeri ya buliidho okusobola okusalawo ebinaava mu kunoonereza kuno. Era olina eidembe okusaba anoonereza okubona ku mawulirego era n’okugatereeza bwekiba kyetagisa. Okukuuma amawulire ag’omuntu kitebwaawo mu iteeka ery’omwezi gwa December nga 8 omwaka 1992 erigema ku kuuma ekyaama

*Protection of your private life*

*Your identity and your participation to this study will be treated strictly confidential. You will not be identified by name or in any other identifying manner in files, results or publication concerning this study. Your identity remains secret since personal information will only be designated by a unique participant number (therefore coded).*

*Your personal information will be processed and analysed electronically (in the computer) or manually in order to determine the results of this study. You also have the right to request the researcher to give you access to your personal information and to correct it if necessary. The protection of personal data is legally established in the Law of December 8, 1992 concerning the protection of private life.*

**Akakiiko ak’empisa**

Okunoonereza kuno kwetegerezeibwa akakiiko k’abakenkufu ku k’eitendekero lya tropical medicine

*Ethics committee*

*This study has been reviewed by the Institute of tropical medicine PhD committee.*

**Abantu ab’ebuuzibwaku singa wabagho ebibuuzoebigema ku kunoonereza**

Bw’olowooza nga ofuniemu okukosebwa okwekuusa ku kunoonereza oba bwoba n’ebibuuzo ebigema ku kunoonereza oba eidembelyo nga eyenhigiremu, osobola okutuukirira, buti, mu kiseera eky’okunoonereza oba nga okunoonereza kuwoire.

***Contact persons in the case of questions concerning the study***

*If you think you have incur damage related to the study or if you have questions concerning the study or your rights as a participant, you can contact, now, during or after the study:*

Study Investigator: EverlynWaweru Telephone: +32 486 74 96 95 or +254 722 996 857

Email: [ewaweru@itg.be](mailto:ewaweru@itg.be)

Study Supervisor: Prof. Bart Criel; Institute of Tropical Medicine; Unit of Equity and Health

Email: [bcriel@itg.be](mailto:bcriel@itg.be)

IRB chairperson: Dr. Suzanne Kiwanuka,

skiwanuka@musph.ac.ug, 256-701-888-163/ 256-312-291-397

**Tool 3i: PATIENT EXIT INTERVIEW QUESTIONNAIRE**

Okutegeera engeri edh’endhidandaba eyetololera ku mulwaire ku mutendera gw’amalwaliro agasokerwaku mu maserengeta ga eirungu lya Sahara-Ensonga ya Uganda

*Understanding patient-centred care approaches at the level of primary health care facilities in sub-Saharan Africa: the case of Uganda*

**(Interviewer to fill in this information before proceeding with interview)**

| 1.0 | Date of interview | DAY [__][__] MONTH [__][__]YEAR[__][__][__][__] |
| --- | --- | --- |
| 1.1 | Interviewer code / Initials | [__][__] |
| 1.2 | Name of facility | ___________________________________ |
| 1.3 | District name |  |

| 1.4 | Waligho omulwaire gw’osobola okubuuza?  *Is there a potential patient to interview?* | YES …………………………………… 1  NO ……………………………………. 2 |  |
| --- | --- | --- | --- |

**Inclusion Questions: Circle as**

| - Bakukozeeku?   ***Have you been attended to?*** | Y/N |
| --- | --- |
| - Otera okuviira mu kitundu kino?   ***Do you normally reside in this area?*** | Y/N |
| - Obaire oviira mu kitundu kino okumala emyezi omukaaga egyibisegho?   ***Have you lived in this area for the last six months?*** | Y/N |
|  |  |
| - Ali kubuzibwa aweza emyaka eikumi n’omunaana n’okuswiika?   ***Is the interviewee 18 years and over?***  ***Mubuuze emyaakagye bwoba tiwekakasa***.  Ask their age if you are not sure.  ***Bwaba taweza myaka ikumi na mukaaga (16), tobuuza.***  **If below 16 years of age, do not interview** | Y/N |

[AKAGHAYIRO AKAMUTOLAMU] BWAAKOBA BBE MU BIMU KU BIBUUZO EBIMULEKAMU:

***TERMINATION CLAUSE (IF THEY ANSWER NO TO ANY OF THE INCLUSION QUESTIONS:***

Webale kubaagho okuba nga tusobola okwogeraku niighe; aye olwa leero,titwiidha kweyongera kukubuuza bibuuzo bindi kubanga***(Insert reason depending on which inclusion question was given a no response) Thank you for availing yourself so that we can talk to you; but for today we will not ask you any more questions because of (Insert reason depending on which inclusion question was given a no response)***

**CONSENT**

**This is to be used together with the Informed consent form and information sheet for patients and caregivers**

| - Amainha gange nhinze__________________ndi kukolera eitendekero lya Makerere university   *My name is __________. I am working for Makerere University* |
| --- |
| - Tuli kukola okunoonereza kuno okwega ku ndowoozayo ku mpeereza y’obwidhandabi obusookerwaku.   *We are conducting this study to learn about your perception on the delivery of primary health care services.* |
| - Tuli kukyaalira amalwaliro munaana okuva mu Iganga era tuli kubuuza abalwaire abawera amakumi asatu mu buli irwaliro.   *We are visiting 8 facilities across Iganga and are interviewing up to thirty patients in each facility.* |
| - Buli mulwaire tuli kubuuza ebibuuzo ebigema empeereza yebafuna, enkolagana n’abakola mu by’obulamusawo n’okusobola obulamu bwaibwe.   *For each patient we are asking questions concerning services they received, their interaction with health care workers and management of their health*. |
| - Okubuuza woonawoona kwiidha kutwala edhakika makumi asatu   *The whole interview will take approximately 30 minutes.* |
| - Titwetaaga kumanha mainhago, era amawulire goonagoona gonatugha gaidha kutwalibwa nga ga kyaama.   *We do not need to know your name, and all information you give us will be treated confidentially.* |
| - Okwenhigira mu kunoonereza kuno kwa kyeyendeire. Bwoikiriza buti me oluvainhuma n’okyuusa ekirowooo, oli waidembe okuvamu esaawa yoonayoona.   *Your participation in this study is voluntary. If you agree now and later change your mind, you are free to withdraw at any time.* |
| - Tubite mu lupapula olusaba olukusa   *Go through the ICF form*   - Otegeire? Olinaku ebibuuzo byonabyona?   *Have you understood? Do you have any questions?* |
| - Kirungi okuja mu maiso n’okubuuza?   *Is it okay that we proceed with the interview?(circle one appropriate response)*  Yes................................. 1  No.................................. 2 |

| 1.5 | OLUKUSA: Nkakasa nti nsomyemu olupapula oluliku obubaka era n’olupapula olusaba olukusa era nainonola okunoonereza kunoeri abuuzibwa nti era ategeera engeri n’omugaso gw’okunoonereza era yaikiriza okwenhigiramu. Awereibwa omukisa okubuuza ebibuuzo ebiiribwamu mu bumativu. Kopi y’ekiwandiiko ekisaba olukusa esigaire n’abuuzibwa n’endagiriro olw’ebibuuzo by’ayinza okuba nanbyo.  *CONSENT: I certify that I have gone through the information sheet and consent form and explained this study to the interviewee and that s/he understands the nature and purpose of the study and consents to participate. S/he has been given an opportunity to ask questions which have been answered satisfactorily. A copy of the consent form has been left with the interviewee with contacts for any questions they may have* | YES ……………………………………………. 1  NO ………………..……………………………. 2  Patient signature or thumbprint |
| --- | --- | --- |
| 1.6 | Esaawa okubuuza yekutandiikireku Time Interview Started. ………………… | HOUR[__][__] MINUTE[__][__] |

**MAIN QUESTIONS(Circle all appropriate responses)**

| Buti nandienze okubuuzaku ebibuuzo bitono ku ighe n’amakaago  ***Now I would like to ask a few questions about you and your family. (circle appropriate response(s))*** | | |
| --- | --- | --- |
| 2.0 | Musaadha oba Mukazi  ***Male or Female*** | Musaadha  Male………………………….................... 1  Mukazi  Female………………………………......... 2 |
| 2.1 | Olina emyaaka emeka?  ***How old are you?*** | WAGATI W’EMYAAKA 16 KU 24…......1  BETWEEN 16-24YRS…............................1  WAGATI W’EMYAAKA 25 KU 44..........2  BETWEEN 25-44YRS…............................2  EMYAAKA 45 N’OKWIIRA WAIGULU..3  45YRS & ABOVE ……..............................3  TIIDHI........…………;;…….…................99  DK ………………………….…................99 |
| 2.2 | Wamaliriza okusoma kwa pulaimale?  ***Did you complete primary school education?*** | YII/YES………………………...........................1  MBE/NO…. ………………………...................2 |
| 2.3 | Wamaliriza okusoma kwa siniya?  ***Did you complete secondary school education?*** | YII/YES………………………...........................1  MBE/NO…. ………………………...................2 |
| 2.4 | Osobola okusoma embaluwa eghandikiibwa mu luzungu?  ***Can you read a letter written in English?*** | YII/YES………………………...........................1  MBE/NO…. ………………………...................2 |

| 2.5 | | Lwaki oidhe okukyaalira eirwaliro olwa leero?***Why did you visit the facility today?***   1. Mweene mulwaire   ***SICK THEMSELVES***   1. Kuleeta mwaana mulwaire   *BRINGING SICK CHILD(<16 YRS )*   1. Kuleeta mulwaire mukulu   *BRINGING SICK ADULT*   1. Kwidhandaba maama na mwaana   *MCH*   1. Entegeka y’eizaire (FP)   Family planning  *FP*   1. Kunwa bulezi/kukeberebwa nga mmaze okuzaala   *ANC/PNC*   1. Akalwaliro akalimu buli kimu ku ndabirira ey’abalwaire   *ART clinic*   1. *Kukeberebwa/kuwandikirwa*   *EXAMINATION/ PRESCRIPTION*   1. *Ekindi OTHER (SPECIFY)* | | YII/*YES* MBE/*NO*  1 2  1 2  1 2  1 2  1 2  1 2  1 2  1 2  1 2  ________________________________________ | | |
| --- | --- | --- | --- | --- | --- | --- |
| 2.6 | | Gunno n’omulundi gwo ogusooka okwidha kwiirwaliro?  ***Is this your first visit to the facility?*** | | YII/YES……………………….................... 1  MBE/NO…........………….......................... 2 | | |
| 2.7 | | Wano n’ekifo ekisinga kuba kya kumpi mu waidhandabirwa awasooka?  ***Is this the nearest primary care facility to your area of residence?*** | | YII/YES……………………….................... 1  MBE/NO…........………….......................... 2 | | |
| 2.8 | | Lwaki wasazeewo okwiidha ku irwaliro lino?  ***Why did you choose to come to this facility?*** *(select all that apply)*? *(Londaku ebyo byoonabyoona ebikola)* | | N’eriri okumpi ni waka ewange  ***It is the nearest to my home………….. 1***  Lwa bwerere  ***It is free ………………………………. 2***  Obulungi bw’omutindo ogw’empeereza  ***Good quality of care experience ……... 3***  Nnenda abasawo  ***I like the clinician(s) …………………. 4***  Bansindika wano  ***I was referred here …………………… 5*** | | |
| 2.9 | | Kikutwaliire ibanga ki (mu saawa) okwidha ku irwaliro era na ntambulaki?  ***How long (in hours) did it take you to come to the facility and by which means?*** | |  | | |
| Buti nandienze okubuuzaku ebibuzo ebigema ku gha w’ofuna obwidhandhabi  ***Now I would like to ask you some questions concerning where you get health care*** | | | | | | |
| 2.10 | | Bwoba n’ekizibu ku bulamu, ogyaawa?  ***When you have a new health problem, where do you go?*** | | Akalwaliro akasokerwaku***……............................. 1***  ***The primary health care clinic***  Akaduuka akatunda eiddagala***………………….. 2***  ***The local drug shop***  Omwidhandabi owo ku kitundu***………………... 3***  ***The community health worker***  Eirwaliro eikulu mu disitulikiti***…………….…….. 4***  ***The district hospital***  Ekindi (inhonola)…………………………………5  ***Other specify*** | | |
| 2.11 | | Ogya bakukeberaku?  Do you go for medical check-ups? | | YII/YES………………………..........................1  Inhonola gha..................................................  Specify where  MBE/NO……......………….......................... 2 | | |
| 2.12 | | Bwobona omukugu, omusawowo ateekwa okukakasa oba okuwandikira ebaluwa okwongerayo?  ***When you see a specialist, does your doctor have to approve or give you referral?*** | | YII/YES……………………….................... 1  MBE/NO……......………….......................... 2 | | |
| 2.13 | | Omusawo aghandika akabaluwa eri omukugu gwebaba bakukobye?  ***Does the clinician write a note to the recommended specialist?*** | | YII/YES……………………….................... 1  MBE/NO…........………….......................... 2 | | |
| 2.14 | | Ebizuulibwa okuva mu kukyaalira omukugu mubyogeraku n’omusawo wo asokerwaku?  ***Do you discuss the findings from the visit to the specialist with your primary care clinician?*** | | YII/YES……………………….................... 1  MBE/NO…........………….......................... 2 | | |
| ***Buti nandienze okubuuzaku ebibuuzo ebigema ku irwaliro lino. Oyinza obutamanha kya kwiiramu ku byonabyona-otaloba kunkobera eyo bweeba nga n’ensonga. Now I would like to ask you some questions concerning this facility. You might not know all of the answers - do not hesitate to let me know if that is the case.(circle appropriate response)*** | | | | | | |
|  |  | | Olunaku *Day* | | Yii/Yes Mbe/No | Esaawa (nga Ibiri edhokunkyo okutuuka ku Ikumi nandala edh’eiggulo)  Time (e.g. 8am-5pm) |
| 3.0 | Oidhi oba nga eirwaliro liigulwa era saawa imeka mu biseera bino?  ***Do you know if and when the facility is open on:*** | | Ennaku edh’okukola *Weekdays* | | 1 2 |  |
|  |  |  | Weekendi *Weekends* | | 1 2 |  |
|  |  |  | Obwiire  *At night* | | 1 2 |  |
|  |  |  | Ennaku enkulu *Public holidays* | | 1 2 |  |
| 3.1 | Bw’olwala, nga eirwaliro liigule, omuntu ow’agho asobola okukubona ku lunaku lweene olwo?  ***When you get sick, and the facility is open, would someone from there see you the same day?*** | | YII/YES……………………….................... 1  MBE/NO…........………….......................... 2 | | | |
| 3.2 | Bw’olwala, nga eirwaliro liigule, obona omusawo n’omulala oyo buli mulundi?  ***When you get sick, and the facility is open, do you see the same clinician every time?*** | | YII/*YES*……………………….................... 1  MBE/*NO*…........………….......................... 2 | | | |
| 3.3 | Ekifo bwewaba waigale,eriyo ennamba y’eisimu kw’osobola okukuba bwoba olwaire?  ***When the office is closed, is there a phone number you can call when you get sick?*** | | YII/YES……………………….................... 1  MBE/NO……. ………………...................... 2  TAIDHI/DK…………………...................... 99 | | | |
| 3.4 | Bwolwala nga eirwaliro liigale, omuntu ow’agho asobola okukubona ku lunaku lweene olwo?  ***When the facility is closed and you get sick, would someone there see you the same day?*** | | YII/YES……………………….................... 1  MBE/NO……. ………………...................... 2  TAIDHI/DK…………………...................... 99 | | | |
| 3.5 | Osobola okutuukirira/okusaba omusawo gwoyenda ku irwaliro?  ***Are you able to access/request a specific clinician at the facility?*** | | YII/*YES*……………………….................... 1  MBE/*NO*…........………….......................... 2 | | | |
| 3.6 | Osobola okutuukirira omusawo gwoyenda ku issimu?  ***Are you able to access a specific clinician by phone?*** | | YII/*YES*……………………….................... 1  MBE/*NO*…........………….......................... 2 | | | |
| 3.7 | Waali owulireku ebibiina ebiwanirira abalwaire?  ***Have you ever heard of any patient support groups?*** | | YII/*YES*……………………........................ 1  Inhonola gha / *If yes specify*………………….  MBE/*NO*……. ……………………................. 2 | | | |
| 3.8 | Oidhiku omuntu yenayena oba omukiise mu bibiina ebiwanirira abalwaire?  ***Do you know any patient support groups members or representatives?*** | | YII/*YES*……………………….................... 1  MBE/*NO*…........………….......................... 2 | | | |

| Buti nandienze okubuuzaku ebibuuzo ebitono ku byobiseemu wano ku irwaliro olwa leero.  ***Now I would like to ask a few questions about your experience at the facility today (circle all appropriate responses)*** | | | | | | |
| --- | --- | --- | --- | --- | --- | --- |
| 4.0 | Ani gwemuwayiiya naye ku irwaliro olwa leero?  ***Who did you interact with at the facility today? (insert number of staff)*** | Waiting bay__________________________________  Registration__________________________________  Consultation room_____________________________  Laboratory___________________________________  Drug dispenser________________________________  Other area____________________________________ | | | | |
| 4.1 | Olinze kumala ibangaki nga okaali:  ***How long did you wait before:***  ***(Wandiika omuwendo gwa saawa n’edakiika mu kifo ekitereibwaawo) (Write the number of hours and minutes in the space provided)*** | Kubona dokita.........................[__] Hours [__] Minutes  ***Seeing the doctor***  Kufuna biviire mu kukebera…[__] Hours [__] Minutes***Getting lab results***  Kufuna idagala.……..............[__] Hours [__] Minutes  ***Getting medication***  Omugaite gw’ekiseera ku irwaliro[__] Hours [__]***Minutes***  ***Total time at the facility*** | | | | |
| Mu kino kitundu, nandienze okwiizaku einhuma ku kuwayakwo n’omusawo leero era ote ebigambo bino wammanga ku idaalaokuva ku kwiikiririza irala okutuuka ku butaikiririza irala.***For this section, I would like you to look back at your interaction with the clinician today and rate the following statements ranging from strongly agree to strongly disagree (circle one appropriate response)***  **Sources:***(Mead and Bower 2002, Stewart 2003, Hudon, Fortin et al. 2011)* | | | | | | |
| **4.2** Okwekenenia obulamu, obulwaire, ni by’obyitamu mu kulwala: Omutendera ogw’okwebuzaaku  ***Exploring health, disease and the illness experience: Consultation process*** | | **Ndikiririza irala Strongly Agree** | **Ndikiriza Agree** | **Tasalawo/ali awo wagati Undecided**  **/ Neutral** | **Taikiriza Disagree** | **Taikiririza irala Strongly Disagree** |
| Omusawo anamwisa mu ngeri endeteire okuwulira obulungi  ***The health worker greeted me in a way that made me feel comfortable*** | | 5 | 4 | 3 | 2 | 1 |
| Omusawo akozeisa olulimi lwensobola okutegeera  ***The health worker used a language that I could understand*** | | 5 | 4 | 3 | 2 | 1 |
| Ekifo aweebuuzibwaamu kibaire kya kyaama era nga kirungi  ***The consultation space was private and comfortable*** | | 5 | 4 | 3 | 2 | 1 |
| Omusawo ankubiriza okugha ebirowoozo byange ebigema ku bulamu/bulwaire bwange.  ***The health worker encouraged me to express my thoughts concerning my health/illness*** | | 5 | 4 | 3 | 2 | 1 |
| Nsoboire okwogera ku nsonga dhange edhindeese olwa leero  ***I was able to discuss my reasons for coming today*** | | 5 | 4 | 3 | 2 | 1 |
| Nkubagainia ebirowoozo lwaki kibaire kya mugaso nze okwiidha ku irwaliro (nga obutasigala waka oba obutagya ku musawo wa kirugavu)  ***I discussed why it was important for me to come to the facility (i.e. and not stay at home or visit chemist…)*** | | 5 | 4 | 3 | 2 | 1 |
| Omusawo abaire wa kisa  ***The health worker was sympathetic*** | | 5 | 4 | 3 | 2 | 1 |
| Omusawo awuliriza bulungyi era kyendowoziiza nti n’embeera/ekizibukimunoonie.  ***The health worker listened carefully and was interested in what I thought the situation / problem was*** | | 5 | 4 | 3 | 2 | 1 |
| Nsoboire okwinhonola obubonero bwange  ***I was able to describe my symptoms*** | | 5 | 4 | 3 | 2 | 1 |
| Omusawo awuliriza bulungyi erakimunoonie bwendogeire ku bubonero bwange.  ***The health worker listened carefully and was interested when I talked about my symptoms*** | | 5 | 4 | 3 | 2 | 1 |
| Omusawo ambuziiza ku ki kyendowooza nga n’ekiri kuviiraku obubonero bwange.***The health worker asked me what I believe is causing my medical symptoms*** | | 5 | 4 | 3 | 2 | 1 |
| Omusawo ayenze inho okumanha obwidhandabi bwenafunaku einhuma  ***The health worker was interested in what treatment I had before*** | | 5 | 4 | 3 | 2 | 1 |
| Nsoboire okwinhonolaowbidhandhabi bwenabaire nfunieku e’inhuma  ***I was able to explain treatment that I had got before*** | | 5 | 4 | 3 | 2 | 1 |
| Omusawo asoboire okulinga mu biwandiiko byange eby’obulamu era yambuuza ebibuuzo  ***The health worker was able to look back at my health records and ask questions*** | | 5 | 4 | 3 | 2 | 1 |
| Omusawo ayenze inho okumanha ki kyenayenze kikolebwe  ***The health worker was interested in what I wanted to be done*** | | 5 | 4 | 3 | 2 | 1 |
| Ndizeemu ebibuuzo by’omusawo byonabyona mu bulambulukufu  ***I answered all the health worker’s questions honestly*** | | 5 | 4 | 3 | 2 | 1 |
| Omusawo ategeire kyembaire ow’okwogera  ***The health worker understood what I had to say*** | | 5 | 4 | 3 | 2 | 1 |
| **4.3 Okwekeeneenia n’endidhandaba**  ***Diagnosis and treatment***  ***(circle one appropriate response)***  *Sources: (Mead and Bower 2002, Stewart 2003, Hudon, Fortin et al. 2011)* | | **Ndikiririza irala Strongly Agree** | **Ndikiriza Agree** | **Tasalawo/ali awo wagati Undecided**  **/ Neutral** | **Taikiriza Disagree** | **Taikiririza irala Strongly Disagree** |
| Singa kyetagisa okukebera omubiri, omusawo ainhonoire bulungi ekikoleibwa na lwaki?  ***If a physical examination was required, the health worker fully explained what was done and why*** | | 5 | 4 | 3 | 2 | 1 |
| Singa kyetagisa,omusawo akwinhonoire okukeberwa okwetagisa okuzuula ekizibu  ***If required the health worker explained the lab tests needed to explore the problem*** | | 5 | 4 | 3 | 2 | 1 |
| Omusawo ainhonoire ebiviire mu kukeberebwa  ***The health worker explained the results of the lab tests*** | | 5 | 4 | 3 | 2 | 1 |
| Omusawo ainhonoire ekizibu n’ekirigha  ***The health worker explained what the problem was*** | | 5 | 4 | 3 | 2 | 1 |
| Omusawo ainhonoire kiki (biki)ebivaaku ekizibu  ***The health worker explained what the cause(s) of the problem was (were)*** | | 5 | 4 | 3 | 2 | 1 |
| Omusawo atesiiza ninzeeby’okulondaku ku ndidhandaba  ***The health worker discussed treatment options with me*** | | 5 | 4 | 3 | 2 | 1 |
| Omusawo ankobeire egya okuba endhidandaba /obwidhandabi  ***The health worker told me what the treatment / medication would do*** | | 5 | 4 | 3 | 2 | 1 |
| Omusawo ainhonoire obuzibu obuyinza okuva mu ndhidandaba /obwidhandabi  ***The health worker explained treatment/ medication side effects*** | | 5 | 4 | 3 | 2 | 1 |
| Omusawo ampaire amawulire agamala nga bwenkyenze  ***The health worker gave me as much information as I wanted*** | | 5 | 4 | 3 | 2 | 1 |
| Omusawo ankubiriza okubuuza ebibuuzo  ***The health worker encouraged me to ask questions*** | | 5 | 4 | 3 | 2 | 1 |
| Mpuliire bulungi okubuuza ebibuuzo  ***I felt comfortable to ask questions*** | | 5 | 4 | 3 | 2 | 1 |
| Omusawo aizemu ebibuuzo n’ensonga dhange  ***The health worker responded to my questions and concerns*** | | 5 | 4 | 3 | 2 | 1 |
| Tukubagainia ebirowoozo n’omusawo ku mitendera egyiiraku, nga mwotaire okulondoola n’okukyaala okwiiraku  ***The health worker discussed next steps, including follow-up plans and next visits*** | | 5 | 4 | 3 | 2 | 1 |
| Omusawo akebeire okukakasa nga ntegeire buli kintu  ***The health worker checked to be sure I understood everything*** | | 5 | 4 | 3 | 2 | 1 |
| Omusawo akebeire okukakasa nga entegeka y’obwidhandabi nnangu gyendi  ***The health worker checked to be sure the treatment plan was manageable for me*** | | 5 | 4 | 3 | 2 | 1 |
| Omusawo ainhonoire ebiyinza okuva mu mu kizibu/mbeera ey’obulamu bwange yebwiidhaku  ***The health worker explained the long term consequences of my medical problem / condition*** | | 5 | 4 | 3 | 2 | 1 |
| The health worker treated me with respect | | 5 | 4 | 3 | 2 | 1 |
| **4.4 Okutegeera omuntu yenayena: Ekisa, obwesigwa, era n’ekinonereze kyekita ku bulamu**  ***Understanding the whole person: Empathy, trust and interest of effect on life***  ***(circle one appropriate response)***  *Sources: (Mead and Bower 2002, Stewart 2003, Hudon, Fortin et al. 2011)* | | **Ndikiririza irala Strongly Agree** | **Ndikiriza Agree** | **Tasalawo/ali awo wagati Undecided**  **/ Neutral** | **Taikiriza Disagree** | **Taikiririza irala Strongly Disagree** |
| Omusawoyalaze nga anfaakuera yandabirira nga omuntu  ***The health worker showed care and concern about me as a person*** | | 5 | 4 | 3 | 2 | 1 |
| Omusawo mutairemu ekitiibwa era na mwiikiriza nga omuntu  ***I showed the health worker respect and accepted them as a person*** | | 5 | 4 | 3 | 2 | 1 |
| Omusawo andeteire okuwulira obutebenkevu  ***The health worker made me feel at ease*** | | 5 | 4 | 3 | 2 | 1 |
| Omusawo alinze inho kubuzibu obulwaire/embeera bweeta ku maka gange oba ku bulamu bwange  ***The health worker was interested in the effect of the problem/condition on my family or personal life*** | | 5 | 4 | 3 | 2 | 1 |
| Omusawo alinze inho kubuzibu obulwaire/embeera bweeta ku mirimu gyange egya buliidho  ***The health worker was interested in the effect of the problem/condition on everyday activities*** | | 5 | 4 | 3 | 2 | 1 |
| Omusawo aikiriza ku li ekizibu lwekirimalwaagho oba okwiikaikanizibwa  ***The health worker was positive about when the problem would be solved or settled*** | | 5 | 4 | 3 | 2 | 1 |
| Omusawo antaamu ekitiibwa  ***The health worker respects me*** | | 5 | 4 | 3 | 2 | 1 |
| Nnesiga omusawo okukola okusalawo okulungi ku ndabirira ey’obulamu bwange  ***I trust the health worker to make good decisions about my health care*** | | 5 | 4 | 3 | 2 | 1 |
| Omusawo ono afa inho ku muwendo okusingaku n’ogwo ogwetagibwa ku bulamu bwange  ***This health worker cares more about the cost than what is needed for my health*** | | 5 | 4 | 3 | 2 | 1 |
| Omusawo ono ankoberanga amazima ku bulamu bwange, ni bwekiba nti amawulire mabi  ***This health worker would always tell me the truth about my health, even if it was bad news*** | | 5 | 4 | 3 | 2 | 1 |
| Singa ensobi yakolebwa mu idagala lyange, omusawo wange agezaaku okunkweka ensobi  ***If a mistake was made in my treatment, my health worker would try to hide it from me*** | | 5 | 4 | 3 | 2 | 1 |
| **4.5 Obwesigwa n’obumativubw’omulwaire Patient confidence and satisfaction**  ***(circle one appropriate response)***  *(Mead and Bower 2002, Stewart 2003, Van Eygen, Van Lerberghe et al. 2007, Hudon, Fortin et al. 2011)* | | **Ndikiririza irala**  ***Very confident*** | **Ndikiriza Agree**  ***Somewhat confident*** | **Tasalawo/ali awo wagati *Undecided***  ***/ Neutral*** | **Taikiriza *Not confident*** | **Taikiririza irala**  ***Not confident at all*** |
| Ndi mumativu inho n’okukyalira kwange eri omusawo  ***I am totally satisfied with my visit to the health worker*** | | 5 | 4 | 3 | 2 | 1 |
| Omusawo ono kwaga asobola okukola ku buli kizibu kyonakyona eky’obulamu kyenhinza okuba nakyo  ***This health worker can take care of almost any medical problem I might have*** | | 5 | 4 | 3 | 2 | 1 |
| Nsobola okwidha eri omusawo ono okuyambibwa mu kizibu ekigema ku nze oba ku mbeera ey’obwongo  ***I could go to this health worker for help with a personal or emotional problem*** | | 5 | 4 | 3 | 2 | 1 |
| Ndi n’okwiikiriza nti omusawo andidhi n’ebyafaayo byange  ***I’m confident that the health worker knows me and my history*** | | 5 | 4 | 3 | 2 | 1 |
| Ndi mwekakafu nti omusawo ategeera ebirowoozo byange  ***I’m confident that the health worker understands my emotion needs*** | | 5 | 4 | 3 | 2 | 1 |
| Ndi n’okwiikiriza nti omusawo ategeera okusalagho kwange mu by’obughangwa n’omwoyo  ***I’m confident that the health worker understands my cultural and spiritual preferences*** | | 5 | 4 | 3 | 2 | 1 |
| Ndi n’okwiikiriza nti omusawo aidhi ku buvunanhizibwa bwange mu maka, ku mulimu oba ku issomero  ***I’m confident that the health worker knows about my responsibilities at home, work or school*** | | 5 | 4 | 3 | 2 | 1 |
| Ndi mutebenkevu okubuuza ebibuuzo ebingemaku  ***I’m comfortable asking personal questions*** | | 5 | 4 | 3 | 2 | 1 |
| Ntegeera obwidhandabi/entegeka ey’eidagala  ***I understand the treatment / medical plan*** | | 5 | 4 | 3 | 2 | 1 |
| Nsobola okwinhonola eidagala lyendikumira  ***I can explain the medicines I am taking*** | | 5 | 4 | 3 | 2 | 1 |
| Ntegeera ebiragiro eby’omusawo  ***I understand the health worker’s directions*** | | 5 | 4 | 3 | 2 | 1 |
| Nnina enkyuukakyuuka ennungi yenesubira mu bulamu bwange  ***I have a good idea about the changes to expect in my health*** | | 5 | 4 | 3 | 2 | 1 |
| Nsobola okutegeera engeri obwidhandabi bwange gyebuli kutambula  ***I am able to understand how my treatment is going*** | | 5 | 4 | 3 | 2 | 1 |
| Mpulira nga omusawo ono ambisiiza bundi olw’e ighanga lyange.  I feel this health worker treated me differently because of my ethnicity | | 5 | 4 | 3 | 2 | 1 |
| Mpulira nga omusawo ono ambisiiza mu ngeri ey’endhawulo olw’obwegerese bwange  ***I feel this health worker treated me differently because of my level of education*** | | 5 | 4 | 3 | 2 | 1 |
| Mpulira nga omusawo ono ambisiiza mu ngeri ya ndawulo olw’obusobozi bwange mu kusasula  ***I feel this health worker treated me differently because of my ability to pay*** | | 5 | 4 | 3 | 2 | 1 |
| **Okwegeresa n’okutumbula eby’obulamu Health education and promotion**  ***(circle one appropriate response)***  **Sources:***(Mead and Bower 2002, Stewart 2003, Hudon, Fortin et al. 2011)* | | **Ndikiririza irala Strongly Agree** | **Ndikiriza Agree** | **Tasalawo/ali awo wagati Undecided**  **/ Neutral** | **Taikiriza Disagree** | **Taikiririza irala Strongly Disagree** |
| Omusawo ayogeire ku ngeri edh’okukendeeza ku mikisa egy’okufuna obulwaire yebwiidha  ***The health worker talked about ways to lower the risk of future illness*** | | 5 | 4 | 3 | 2 | 1 |
| Ampaire amagezi ku ngeri ey’okuziyizaamu ebizibu eby’obulamu mu yebwiidha (sukaali, emizze emirungi mu bulamu, okukola duyiro, okwegandaga okukalamu nga kigiira ku bulwaire oba embeera…)  ***Advised me how to prevent future health problems (diet, health habits mentioned exercise, safe sex as relevant to illness or condition…)*** | | 5 | 4 | 3 | 2 | 1 |
| **4.6** Okwongerayokw’okulabirira  ***Continuity of care: referrals***  ***(circle one appropriate response)***  **Sources:** *(Mead and Bower 2002, Stewart 2003, Hudon, Fortin et al. 2011)* | | **Ndikiririza irala Strongly Agree** | **Ndikiriza Agree** | **Tasalawo/ali awo wagati Undecided**  **/ Neutral** | **Taikiriza Disagree** | **Taikiririza irala Strongly Disagree** |
| Omusawo ono aidhi li okukeberebwa kwange lwekutuuka  ***This health worker knows when I am due for a check up*** | | 5 | 4 | 3 | 2 | 1 |
| Omusawo ono buli kiseera alondoola ekizibu kyenali nakyo bwenkyaala ku mulundi ogwiiraku oba akuba eisimu  ***This health worker always follows up on a problem I had at the next visit or by phone*** | | 5 | 4 | 3 | 2 | 1 |
| Omusawo ono buli kiseera anondoola ku kukyaala okwiiraku n’abasawo abandi  ***This health worker always follows up on the next visit with other health workers*** | | 5 | 4 | 3 | 2 | 1 |
| Omusawo ono anamba okukola obulagane bwemba nnenda okubona omukugu/omusawo ku irwaliro erririku waigulu  ***This health worker tells me when I need to see a specialist*** | | 5 | 4 | 3 | 2 | 1 |
| Omusawo ono anamba okukola obulagane bwemba nnenda okubona omukugu/omusawo ku irwaliro erririku waigulu  ***This health worker helps me to book appointments when I need to see a specialist/ health worker in a higher level facility*** | | 5 | 4 | 3 | 2 | 1 |
| Omusawo ono ayogera n’abasawo bale abandi bembona  ***This health worker communicates with the other health providers I see*** | | 5 | 4 | 3 | 2 | 1 |
| Omusawo ono aidhi ebyaviire mu kukeberebwa bwenakyaalire omukugu  ***This health worker knows what the results of the specialist visit were*** | | 5 | 4 | 3 | 2 | 1 |
| Nga maze okukyaalira omukugu oba okufuna empereza ey’endhawulo, omusawo ono ayogeire niiwe ku kyabaire mu kukyaala  ***After going to the specialist or special service, this health worker talked with you about what happened at the visit*** | | 5 | 4 | 3 | 2 | 1 |
| Omusawo wo aboneike nga ayenze inho omutindo ogw’empereza gw’ofuna okuva ku mukugu oyo oba empereza ey’endhawulo  ***Your health worker seemed interested in the quality of care you get from that specialist or special service*** | | 5 | 4 | 3 | 2 | 1 |
| **4.7** Okukola okusalawo okw’aghalala  **Joint Decision Making**  ***(circle one appropriate response)***  **Sources:** *(Mead and Bower 2002, Stewart 2003, Hudon, Fortin et al. 2011)* | | **Ndikiririza irala Strongly Agree** | **Ndikiriza Agree** | **Tasalawo/ali awo wagati Undecided**  **/ Neutral** | **Taikiriza Disagree** | **Taikiririza irala Strongly Disagree** |
| Omusawo yanhigire mu kusalawo nga bwenakyenze  ***The health worker involved me in decisions as much as I wanted*** | | 5 | 4 | 3 | 2 | 1 |
| Twogeire era twembi twaikirizagania ku kizibu n’ekirigha  ***We discussed and together agreed on what the problem was*** | | 5 | 4 | 3 | 2 | 1 |
| Tuviireyo n’ekigendererwa eky’obwidhandabi/enteekateeka y’obwidhandabi  ***We came up with the goals of treatment / health care plan*** | | 5 | 4 | 3 | 2 | 1 |
| Tutesiiza ku mitendera egyiiraku omuli n’entegeka edh’okundutaku  ***We discussed next steps, including any follow-up plans*** | | 5 | 4 | 3 | 2 | 1 |
| Tutesiiza ku buvananhizibwa bwa buli omu (omusawo n’omulwaire) mu ntegeka yange ey’obwidhandabi  ***We discussed our respective roles (the health worker and the patient) in my health care plan*** | | 5 | 4 | 3 | 2 | 1 |
| Omusawo ampaire amawulire gonagona genenze  ***The health worker gave me all the information I need*** | | 5 | 4 | 3 | 2 | 1 |
| Ekiseera kyensoboire okumala n’omusawo ono tikibaire kinene kimala kukola ku buli kimu kyenhenze  ***The time I was able to spend with this nurse was not long enough to deal with everything I wanted*** | | 5 | 4 | 3 | 2 | 1 |
| Mpaire endowooza yange (okwikiriza oba obutaikiriza) ku bika by’okukebera oba obwidhandabi omusawo wange byalagiire  ***I gave my opinion (agreement or disagreement) about the types of tests or treatment that my health worker ordered*** | | 5 | 4 | 3 | 2 | 1 |
| Omusawo yagemagainia okubusabuusa kwonakwona ku kukebera oba obwidhandabi ebyasalibwaawo  ***The health worker handled any doubts about the tests or treatment that were recommended*** | | 5 | 4 | 3 | 2 | 1 |
| Omusawo yankubiriza okutwaala obuvunanhizibwa bwenenda mu kulabirirwa kwange  ***The health worker encouraged me to take the role I wanted in my own care*** | | 5 | 4 | 3 | 2 | 1 |
| Omusawo akuleka waalinga ku biwandiikobyo eby’obulamu  ***The health worker lets you look at your medical records*** | | 5 | 4 | 3 | 2 | 1 |
| **4.8** Ebiva mu kwidhandabwa: mu kitundu kino, nandienze olowoze ku ngeri obumanhirivubwo ku irwaliro gyebukoze ku bulamubwo.  **Outcomes of care: *for this section, I would like you to think about how your experience at the facility has affected your health(circle one appropriate response)*** | | | | | | |
| **Sources:** *(Mead and Bower 2002, Stewart 2003, Hudon, Fortin et al. 2011)* | | **Ndikiririza irala Strongly Agree** | **Ndikiriza Agree** | **Tasalawo/ali awo wagati Undecided**  **/ Neutral** | **Taikiriza Disagree** | **Taikiririza irala Strongly Disagree** |
| Obubonero bwange n’obulumi bikendeire  ***My symptoms and pain have reduced*** | | 5 | 4 | 3 | 2 | 1 |
| Kikendeziiza okutya kwange n’okweralikirira  ***It has lessened my fears and anxiety*** | | 5 | 4 | 3 | 2 | 1 |
| Nsobola okwira ku mirimu gyange egya buliidho  ***I am able to return back to my routine activities*** | | 5 | 4 | 3 | 2 | 1 |
| Nsobola okubaaku nikyenkola singa embeera y’obulamu bwange eyononekamuuku (ndidi ow’okutukirira, eky’okukola)  ***I am able to react if my health deteriorates***  ***(I know who to contact, what to do)*** | | 5 | 4 | 3 | 2 | 1 |
| Ndimu amaani okugiira ku idagala eriwandiikibwa  ***I am motivated to follow the treatment prescribed*** | | 5 | 4 | 3 | 2 | 1 |
| ***Which advice do you find difficult to follow?*** | |  | | | | |
| Ekisobozesa omulwaire  ***Patient enablement instrument*(Van Eygen, Van Lerberghe et al. 2007)** | | Okusinziira ku kukyaalakwo eri omusawo leero, owulira nga oli?  ***As a result of your visit to the nurse today do you feel you are?*** | | | | |
|  |  | Bulungi inho  ***Much better*** | Bulungiku  ***better*** | **N’ekirala**  **same** | N’ekirala oba katono  ***Same or less*** | Ghazira waire  ***Not at all*** |
| Osobola okugumira obulamu?  ***Able to cope with life?*** | | 5 | 4 | 3 | 2 | 1 |
| Osobola okutegeera obulwairebwo?  ***Able to understand your illness?*** | | 5 | 4 | 3 | 2 | 1 |
| Osobola okugumira obulwairebwo?  ***Able to cope with your illness?*** | | 5 | 4 | 3 | 2 | 1 |
| Osobola okwekuuma nga oli mulamu?  ***Able to keep yourself healthy?*** | | 5 | 4 | 3 | 2 | 1 |
| Oli mwekakafu ku bulamubwo?  ***Confident about your health?*** | | 5 | 4 | 3 | 2 | 1 |
| Osobola okweyamba?  ***Able to help yourself?*** | | 5 | 4 | 3 | 2 | 1 |
| Overall satisfaction | | | | | | |
|  | | ***Very satisfied*** | ***satisfied*** | ***indifferent*** | ***Not satisfied*** | ***Not satisfied at all*** |
| Number of health workers | | 5 | 4 | 3 | 2 | 1 |
| How health workers communicated with you | | 5 | 4 | 3 | 2 | 1 |
| Your treatment / health plan? | | 5 | 4 | 3 | 2 | 1 |
| Advice / follow-up | | 5 | 4 | 3 | 2 | 1 |
| Will you come back to this facility | | Yes | | No | |  |
| Would you like to see the same health worker on your next visit? | | Yes | | No | |  |
| Would you refer your friend or family to this facility | | Yes | | No | |  |

| 7.0 | Additional questions from the pilot | | |
| --- | --- | --- | --- |
|  | What enables you to participate in discussions and decisions reached together with their health provider? |  | |
|  | What hinders you from participating in discussions and decisions reached together with their health provider? |  | |
|  | Are you a member of any patient support group or organisation? (if yes specify name and type of group of patients) |  | |
|  | During your visit to the facility today did you feel like you were handled differently because of your: circle appropriate response | | |
|  |  | YES | NO |
|  | Condition or illness | 1 | 2 |
|  | Age | 1 | 2 |
|  | Gender | 1 | 2 |
|  | Education | 1 | 2 |
|  | Religion | 1 | 2 |
|  | Ethnicity | 1 | 2 |
|  | Financial ability to pay for the services at the health care facility | 1 | 2 |
|  | If treated differently, what was done differently, why do they feel they were treated differently |  | |
|  | Anoonereza ate wano byayogeraku/byaboine ku kubuuza kuno  ***Field worker to enter any comments/ observations about this interview*** |  | |

Webale inho olw’okwenhigirakwo mu kubuuzibwa kuno. Olinaku ebibuuzo ebindi?

***Thank you very much for your participation in this interview. Do have any questions***?

INTERVIEW ENDED AT HR [__][__] MIN [__][__]

**CHECKED BY:**INTERVIEWER CODE [__][__] DATE ___________

**Tool 3j:FOCUS GROUP DISCUSSIONS INFORMATION SHEET**

Webale inho okutaayo ebiseerabyo okuba n’oluwayo luno niife leero. Amainha gange nhinze__________________anoonereza okuva mu Institute of Tropical Medicine in Antwerp. Okusookera irala, webale inho olw’ebiserabyo okutyama niinze wano buti. Amainha gange nhinze__________________anoonereza okuva mu itendekero lya Tropical Medicine mu Antwerp. ITM n’erimu ku itendekero eritendeka n’okunoonereza nga likolera ghalala n’amatendekero mangi ag’ebya science, gavumenti, n’ebitongole munsi yoonayoona kulw’okutumbula eby’obulamu n’okugema endwaire okw’olubeerera mu mawanga agali kukula.

*Thank you very much for taking your time to have this discussion with us today.My name is _______________a research assistant working for the Institute of Tropical Medicine (ITM) in Antwerp. The ITM is one of the training and research institutions that works with many scientific institutions, governments and organisations all over the world for long-lasting improvement of health care and disease control in developing countries. In Uganda we are working together with Makerere university.*

Tuli kukola okunoonereza ku ngeri edhisobola okukozesebwa okukakasa nga empeereza eweebwa mu malwaliro agasookerwaku eringa inho ku mulwaire; era buzibu na mikisaki byetunasobola okubitamu. Nandienze okubuuzaaku ebibuuzo ebyekuusa ku kino.Nenda otegeere nti ghazira kituufu oba kifu mu buli ky’okoba. Era, okunoonereza kuno tikugya kumanibwa. Kino kitegeeza nti buli ky’okoba tikiidha kukunonenkerezebwaku. Okunoonereza kwidha kutwala kitundu kya saawa. Kinaaba kirungi singa mpandiika byonandiramu? Nga bwobona, nnina olupapula n’ekalamu okuwandiika, kino kidha kunnamba okwiidhukira. Kansubire nga tofaayo.

*We are conducting a research study looking at approaches that can be taken to ensure the care provided at primary health care facilities is more focussed on the patient; and what challenges and opportunities would be experienced. I would like to ask you some questions in regard to this. I would just like you to know that there is no right or wrong in anything you say. Also, this discussion is anonymous. This means that anything you say will not be traceable back to you. The discussion will take about an hour. Would it be okay if I wrote your answers down? As you can see, I have some paper and pencil to write, this will help me to remember. I hope you do not mind.*

Nga okaali kusalagho kwenhigira mu kunoonereza kuno, kyamugaso okusoma olupapula luno. Olina eidembe okubuuza ebibuuzo ekiseera kyonaakyona. Wandienze mbitte mu lupapula luno niighe? ***[if yes, Continue with this informed consent form, if no terminate the conversation here and record refusal]***

*Before you decide to participate in this study, it is important that you read the information in this form. You have the right to ask questions at any time. Would you like me to go through the form with you?[If yes, Continue with this informed consent form, if no, give them the form to read for themselves and in cases of refusal to participate, terminate the conversation here and record refusal]*

**Omugaso n’okwinhonola ku kunoonereza**

Kuno n’okunoonereza okuzuula emikisa n’obuzibu mu kutuusa endabirira eyetololera ku mulwaire mu malwaliro agasookerwaku mu Uganda. Twidha kuba nga twogera n’abalwaire, abakola mu by’obulamu (abasawo n’abakola egy’okuwereza abantu), abakola amateeka, abasomesa eby’obulamu era n’abanoonereza abenhigira mu malwaliro munaana mu Uganda. N’olwekyo, buli gwekigemaku aidha kwetebwa okwenhigira mu kutegeka n’okukola engeri edh’okutaasa edhigwana, emitendera egy’okulondoola gyidha kukolebwa okupima enkyuuka etereibwabwo okutaasa ku mutindo ogw’empeereza eweebwa mu bifo ebidhandabirwamu ebya ga gavumenti n’eby’obwanakyeegha ebisokerwaaku.

*Purpose and description of the study*

*This is a study to explore opportunities and challenges in the delivery of patient centered primary health care services in Uganda. We shall be talking with patients, health care workers (doctors, nurses and social workers), policy makers, health educators and researchers involved in primary level care at 8 facilities in Uganda. Consequently, all stakeholders will be invited to participate in the design and implementation of an appropriate intervention follow-up phases will be conducted to assess the impact of interventions on the quality of versatile health care services provided at both public and private primary care facilities*

Bwoikiriza okwenigira mu kunoonereza kuno, oidha kubuuzibwa ebibuuzo kungeri gyowuliramu ku kutabagana kwo n’omusawo wo, era n’obusobozi bwo okukola okusalawo okwaghalala okukuuma obulamu bwo oba okusobola obulwairebwo

*If you accept to participate in this study, you will be asked questions on how you feel about the interaction between you and your health care providers; your perception of the quality of care given at the primary care facilities and your ability to make joint decisions to maintain your health or manage your illness.*

**Okutegeera okukeberwa mu kunonerera**

Bwoikiriza okwenhigira mu kunoonereza kuno, ezira kukeberebwa kwa ku mubiri kuja kukolebwa. Bwoba toyenda kwiiramu ekimu ku bibuuzo, osobola okwogera era ndidha kujja ku kibuuzo ekiiraku.

*Examinations in the context of the study*

*If you accept to participate in the discussion, no invasive tests and examinations will be performed. If you do not want to answer any of the questions, you may say so and I will move on to the next question.*

**Okwenhigiramu okwekyeyendeire**

Wenhigiramu kyeyendeire mu kunoonereza kuno era olina eidembe okudhema okwenhigira mu kunoonereza kuno. Okusalawokwo okwenhigira mu kunoonereza kuno oba bbe, tikwiidha kuba na kyamaanhi kyekukola ku bwidhandabi bw’ofuna ku irwaliro. Era olina eidembe okulekera okwenhigira mu kunoonereza kuno ku kiseera kyonakyona, nibwooba oikiriza.

*Voluntary participation*

*You participate entirely voluntarily in this study and you have the right to refuse to participate in the study. Your decision to participate in this study or not, will have no influence whatsoever on the care you get at the facility. You also have the right to stop you participation in the study at any time, even after you have given consent.*

**Obuzibu n’obukaluubirivu**

Tighaabe bukaluubirivu okwenhigira mu kunoonereza kuno.

*Risks and inconveniences*

*There will be no physical risks to participating in this study.*

**Ebirungi**

Titusobola kukakasa nti ng’omuntu oidha kuganulwa mu buligho okuva mu kwenhigira mu kunoonereza kuno leero. Bwoikiriza okwenhigira mu kunoonereza kuno, amawulire okuva mu kunoonereza kuno gasobola okuyamba mu kwongera ku amagezi okuva mu kwogerezagania wagatiwo n’abalwairebo era n’okuyamba abalwaire abandi yebwiidha.

*Advantages*

*We cannot confirm that you will personally benefit directly from your participation in this study today. If you consent to participate in this study, the information resulting from this study can contribute to better knowledge on the interaction between you and your patients and help other patients in future.*

**Okuliyirwa**

Waliwo okusasulwa entambula okuliwo olw’oluwayo luno leero kubanga okubuuzibwa tikukoleibwa ku irwaliro, omuwendo gwidha kusalibwaagho okugiira ku miwendo gya wano n’olugendo olutambuirwa.

*Compensation*

*There is a transport reimbursement available for this discussion today because the interview was not done at the facility, the amount will be determined by local rates and distance travelled.*

**Okukuuma obulamubwo obw’ekyaama**

Ebikugemaku n’okwenhigirakwo mu kunoonereza kuno biidha kutwalibwa nga bya kyaama. Toidha kutegerebwa ku maina oba mu ngeri eyindi ey’okumanibwa mu mpapula, ebinaava oba ebinafulumizibwa nga bigema ku kunoonereza kuno. Ebikugemaku bisigala nga kyaama kayimba nti amawulire ku muntu agidha kutebwaaku namba eyendhawulo (n’olwekyo gaidha kukweekebwa).

*Protection of your private life*

*Your identity and your participation to this discussion will be treated as strictly confidential. You will not be identified by name or in any other identifying manner in files, results or publication concerning this study. Your identity remains secret since personal information will only be designated by a unique participant number (therefore coded).*

Amawulire agakugemaku gaidha kusengedhebwa era gekeeneeneezebwe n’ebyuuma ( mu kyuuma ki kalimagezi) oba mu ngeri ya buliidho okusobola okusalawo ebinaava mu kunoonereza kuno. Era olina eidembe okusaba anoonereza okubona ku mawulirego era n’okugatereeza bwekiba kyetagisa. Okukuuma amawulire ag’omuntu kitebwaawo mu iteeka ery’omwezi gwa December nga 8 omwaka 1992 erigema ku kuuma ekyaama

*Your personal information will be processed and analysed electronically (in the computer) or manually in order to determine the results of this study. You also have the right to request the researcher to give you access to your personal information and to correct it if necessary. The protection of personal data is legally established in the Law of December 8, 1992 concerning the protection of private life.*

*Era n’olwensonga eno tusaba abantu abaligho mu luwayo luno okuukuma ebiri mu luwayo luno nga bya mu luwayo luno.*

*For this reason we also ask the people present during this discussion to keep the content of the discussion i.e. within this group discussion.*

**Akakiiko ak’empisa**

Okunoonereza kuno kwetegerezeibwa akakiiko ka abakenkufu ab’eitendekero lya tropical medicine

*Ethics committee*

*This study has been reviewed by the Institute of tropical medicine PhD committee.*

**Abantu ab’ebuuzibwaku singa wabagho ebibuuzo ebigema ku kunoonereza**

Bw’olowooza nga ofuniemu okukosebwa okwekuusa ku kunoonereza oba bwoba n’ebibuuzo ebigema ku kunoonereza oba eidembelyo nga eyenhigiremu, osobola okutuukirira, buti, mu kiseera eky’okunoonereza oba nga okunoonereza kuwoire.

*Contact persons in the case of questions concerning the study*

*If you think you have incurred damage related to the study or if you have questions concerning the study or your rights as a participant, you can contact, now, during or after the study:*

*Study Investigator: EverlynWaweru Telephone: +32 486 74 96 95 or +254 722 996 857*

*Email:* [*ewaweru@itg.be*](mailto:ewaweru@itg.be)

*Study Supervisor: Prof. Bart Criel; Institute of Tropical Medicine; Unit of Equity and Health*

*Email:* [*bcriel@itg.be*](mailto:bcriel@itg.be)

*IRB chairperson: Dr.suzannekiwanuka, skiwanuka@musph.ac.ug, 256-701-888-163/ 256-312-291-397*

*Tool 3k: FOCUS GROUP DISCUSSIONS – GROUP CONSENT*

**Omutwe ogww’okunoonereza***:* Okutegeera engeri edh’endhidandaba eyetololera ku mulwaire ku mutendera gw’amalwaliro agasookerwaku mu maserengeta ga eirungu lya Sahara-Ensonga ya Uganda

*Understanding patient-centred care approaches at the level of primary health care facilities in sub-Saharan Africa: the case of Uganda*

| *Date of discussion:* | *Moderator:* |
| --- | --- |
| *Venue:* | *Note-taker:* |
| *Time start:* | *No. Participants at start:* |
| *Time stop:* | *No. Participants at stop:* |

*Written Consent for Focus Group Discussions*

Okunoonereza ku twinhonoleibwa______________________________________________okuva mu_____________________________. Tutegeire ebyo byonabyona ebisomeibwa/ebinonoreibwa. Tuweirebwa omukisa okusaba okutangazibwamu era ebibuuzo byaife byairibwamu mu kusiima.

Wano:

1) Tugha olukusa/titugha lukusa* okwenhigira mu kunoonereza kuno

2) Tugha olukusa/titugha lukusa* okuba nga olutambi olw’okubuuza lugemebwa mu maloboozi

(Delete as appropriate)

Tukitegeera nga tusobola okukyuusa endowooza yaife ku mutendera gwonagwona era nga tikiidha kutukosa mu ngeri yoonayoona

Omukono ogw’omukise w’ekibindha_______________________olunaku___________________

Name initials _____________________________Facility Name: ______________________

*We have had the study explained to us by ____________________from __________________.We have understood all that has been read/explained. We were given the opportunity to seek clarification and had our questions answered satisfactorily.*

*We hereby:*

*1) Give consent / do not give consent* to take part in the study*

*2) Give consent/ do not give consent* to having the interview tape recorded*

*(* Delete as appropriate).*

*We understand that we can change our minds at any stage and it will not affect us in any way.*

*Signature of group representative _______________________ Date: ______________________*

*Or Thumbprint*

*Name initials _____________________________Facility Name: ______________________*

***Participant demographic data***

| ***Participant*** | ***Gender*** | ***Age*** | ***Education (Highest level)*** | ***Occupation*** | ***No. of mins to walk to facility*** | ***Name of facility*** | ***Contact details*** |
| --- | --- | --- | --- | --- | --- | --- | --- |
| ***1*** |  |  |  |  |  |  |  |
| ***2*** |  |  |  |  |  |  |  |
| ***3*** |  |  |  |  |  |  |  |
| ***4*** |  |  |  |  |  |  |  |
| ***5*** |  |  |  |  |  |  |  |
| ***6*** |  |  |  |  |  |  |  |
| ***7*** |  |  |  |  |  |  |  |
| ***8*** |  |  |  |  |  |  |  |
| ***9*** |  |  |  |  |  |  |  |
| ***10*** |  |  |  |  |  |  |  |

**Akubagania by’ayogeraku**

*Moderator remarks*

**Tool 3k: INTERVIEW GUIDE FOR GROUP DISCUSSIONS**

Okutegeera engeri edh’endhidandaba eyetololera ku mulwaire ku mutendera gw’amalwaliro agasookerwaku mu maserengeta ga eirungu lya Sahara-Ensonga ya Uganda

maserengeta ga Africa: Ensonga ya Uganda

*Understanding patient-centred care approaches at the level of primary health care facilities in sub-Saharan Africa: the case of Uganda*

**Akubagania asangaaza, yeyandula era n’okwandula kw’abenhigiremu**

**Facilitators welcome, introduction and introduction to participants**

Tusangaire era mwebale kwewayo kwenhigira mu luwayo luno olw’okukubagania ebidhuubo okw’aghalala. Endowoozayo yamugaso era tussima ebiseerabyo. Buti nandienze okuwa obubaka obusingawo ku luwayo lwaife olwa leero. (bita mu lupapula oluliku obubaka n’olusaba olukusa waigulu) ***Welcome*** *and thank you for volunteering to participate in this focus group. Your point of view is important and we appreciate your time. I would now like to give you some information about our discussion to day (go through the information sheet and consent process above).*

Buti nga buli omu bwali obulungi n’oluwayo, tulina obubaka obundi n’okusaba okundi:

*Now* *that everyone is comfortable with the discussion, we have some more information and some requests*:

- Kikulu okuba nti muntu mulala yenka n’ayogera ku kiseera, buli omu aidha kugheebwa omukisa okwogera. Wayinza okubaawo okusikirizibwa okubukiramu ng’omuntu alikwogera aye tusaba okulinda mpaka nga amaze.
- *It is important that only one person speaks at a time, everyone will be given an opportunity to speak. There may be temptation to jump in when someone is talking but we ask to wait until they have finished.*
- Wazira bidhuubo bituufu oba biffu, era tikikukakataku kwiikiriza nabandi mu kibindha.
- *There are no right or wrong opinions, and you don’t have to Ndikiririza with others in the group*
- Era timuteekwa kwogerera mu ntegeka yakwiiraganwaaku
- *you also don’t have to speak in any particular order*
- (Wa amawulire ku by’okunwa n’ebisenge awawumulirwa)…oli waidembe okufula muuku nga okubagania ebidhuubo kulikuja mu maiso aye tusaba nti okikole mpola
- *(Give information on refreshments and restrooms)…you are free to move out during the discussion but we ask that you do so quietly*
- Olinaku ebibuuzo byonabyona?
- *Do you have any questions?*
- Nkale, leka tutandike
- *Ok, lets begin*

**Twesuyemu**

***Warm up***

Okusookera irala nandienze buli omu yeyandule

*First I would like everyone to introduce themselves*

Ebigema ku muwi w’amawulire omukulu ku lupapula olw’amawulire (era kino kisobola okukolebwa aghalala n’okwandula)

Emyaaka, gha webaviira, eirwaliro lyebajaaku (fill in the demographic data capture form)

**Ekibuuzo ekitandika**

Nandienze okuwaayo edakiika ntono okulowooza ku bumanhirivubwo mu bifo eby’empeereza esookerwaku. Waliwo asanwiike okugabanaku niife kyaidhi?

***Introductory question***

*I would like to give you a few minutes to think about your experiences at primary health care facilities. Is anyone happy to share their experiences*?

Ebibuuzo ebitugalaga

Guiding questions

1. What do you think makes a facility of good quality?
2. Why do you choose to come to this facility?
3. As we begun, you said that one of the things that makes a facility good is _____

Is that available in Busowobi health centre?

1. If private facilities were also free, would you still come to this facility? And why?
2. Are there facilities that are not available at Busowobi health centre that you would like to be introduced?
3. For the services that you cannot get at busowobi, where else do you seek health care?
4. Are health workers available at the facility all the time? All the days of the week?
5. What do you do when the health workers are not available?
6. Now we are going to ask you questions about your experience at Busowobi health centre?
7. Before you go to the health facility, how or what things do you have to prepare?
8. What happens from the time you enter the facility to the time you leave?
9. Waiting time, patient flow, consultation, privacy and discussing personal problems or examination of sensitive areas? How do you feel about other patients being there?
10. What support do you get from the facility after you leave the facility?
11. Follow-up calls, if you get ill before the next appointment, if you have to do a test or get drugs from elsewhere…what happens?
12. What are some of the things that this facility does really well, or that you like about this facility?
13. What do you think can be improved and how? Any complaints or challenges?

Eriyo emirimu egyindi egy’omugaso oba ebikolebwa ebibairewo oba ebiriwwo ebirinawo kyebikoze ku mutindo gwempeereza efunibwa mu malwaliro agasookerwaku?

*Are there any other important activities or events that have happened or are happening that have an impact on the quality of care offered in the primary health care facilities?*

Ki kyewandisiinze kwenda kweega okubita mu mirimu gyaife?

*What would you be interested in learning through our work?*

Omuntu owundi gwetuyinza okwogeraku naye?

*Anyone else we should talk to?*

Wandienze okutegezebwaku ku bibindha mwetugya okunanooniaku okuwaya?

*Would you like to be informed of follow-up group discussions? if so kindly leave your contacts with us*

**Okumaliriza:** Webaze gw’olikubuuza olw’okwenhigiramu era omalirize okubuuza

***Conclusion****: Thank the interviewees for their participation and conclude the interview.*

**Patient semi-structured interview guide (translated during the interview)**

Thank you for welcoming us into your home. As we had introduced ourselves during your visit to the facility, my name is _______________and this is my colleague _________________ and we are working together with Makerere university looking into the relationships between patients and health workers and how to improve the quality of care that you receive at the health facility.

1. Thank you again for agreeing to speak with us today, maybe you could begin by telling us a bit about yourself?
   - Family members and role in the family?
   - How long have you lived in this area?
   - How long have you been going to this facility?
   - Perception of health and illness
2. We would like to know your opinion about the primary health care services available in this area
   - Which facilities are available?
   - Any VHTs
   - Medical camps, campaigns etc.
3. When you or a member of your family gets unwell, who recognises and how do you decide what to do first?
   - Go to the facility
   - By drugs from the shop etc.
   - How is the decision made (who makes the decision and what factors are considered)
4. What about health information messages? Where do you get them? Which avenue is the most frequent?

Questions about facility experience

1. More specifically about the day that you visited the facility (where we met you before), which areas did you go through?
   - Registration
   - Clinician
   - Lab
   - Pharmacy
   - ANC
   - Maternity
   - Home visits
   - Any other areas that you have been to

Probe for each area:

- - 1. What was your experience like?
    2. Who did you interact with (relationship with health workers)?
    3. How were you received?
    4. Were you able to express yourself?- remember to not only ask about physical aspects of health or illness but also ask about psychological and emotional aspects of health and illness
    5. Did you have a good conversation with the musawo? – how was the treatment (Treatment process); were procedures explained, were you able to ask questions, were the questions answered. Are there questions that you wanted to ask but could not? Why?
    6. How did you feel at the end of it? (pay attention to patients’ feeling of involvement in decision making, confidence and patient enablement)

1. Why did you choose to go to that facility?
2. Are there things that you like about your experience at the facility?
3. Are there things that you don’t like about your experience at the facility?
4. If you could change something about the facility what would it be?
5. Are you aware of patient rights?
   1. If yes- what are they and where did you hear about them? If no, explain patient rights as written in the service charter
   2. What do you think about them?
   3. What is the situation with patients’ rights at the health facility that you visited (did you feel your rights were considered?)
6. What do you think are the responsibilities of a patient to a health facility? (ask about community ownership of the facility)
7. Is there anything else you would like to tell us about the facility or the health workers?
8. Do you have any questions for us?

**Thank the interviewee for their time**
