# Supplementary material for: Patient perspectives on interpersonal aspects of healthcare and patient-centeredness at primary health facilities: A mixed methods study in rural Eastern Uganda
Source: PLoS One. 2020 Jul 30;15(7):e0236524. doi: 10.1371/journal.pone.0236524 (PMC7392339; doi:10.1371/journal.pone.0236524)
Supplement: S1 Table — A table showing how the questions incorporated in the tools used in the study were developed from previously validated tools and supporting literature. (DOCX) [file pone.0236524.s002.docx]

**Supplementary file S2. Table:** Patient-centered care measurement instruments used in designing the tool to measure patient perceptions of PCC in Uganda

| **Instrument and source** | **Exploring the health, disease and illness experience** | **Understanding the whole person** | **Finding common ground between the doctor and the patient** | **Prevention and health promotion** | **Building the patient and physician relationship** |
| --- | --- | --- | --- | --- | --- |
| Patient perception of patient centeredness (14 questions)  Stewart et al. [38] | 4 | 1 | 9 | 0 | 0 |
| Consultation care measure (21 questions) Little et al.[42, 43] | 6 | 5 | 7 | 2 | 1 |
| Interpersonal processes of care  (29 questions)  Stewart et al. [38] | 4 | 1 | 8 | 1 | 8 |
| Instrument on doctor patient communication skill (19 questions)  Campbell et al.[44] | 2 | 1 | 10 | 0 | 3 |
| Component primary care instrument (52 questions)  Floke et al.[45, 46] | 5 | 5 | 3 | 2 | 6 |
| PCC in Uganda tool (Questions)  Supplementary file 1 | 17 | 8 | 15 | 2 | 13 |
