# Supplementary material for: Patient perspectives on interpersonal aspects of healthcare and patient-centeredness at primary health facilities: A mixed methods study in rural Eastern Uganda
Source: PLoS One. 2020 Jul 30;15(7):e0236524. doi: 10.1371/journal.pone.0236524 (PMC7392339; doi:10.1371/journal.pone.0236524)
Supplement: S2 Table — A table showing the responses of 300 patients to questions about their experience of PCC, and patient reported outcomes. (DOCX) [file pone.0236524.s003.docx]

**Supplementary file S3 Table:** Patients’ perceptions of PCC dimensions and patient reported outcome measures, in rural eastern Uganda

| N=300, the numbers in the table are presented as the number of patients that responded and the %of the N (with 2 decimal places) | **Strongly disagree** | **Disagree** | **Neutral** | **Agree** | **Strongly agree** |
| --- | --- | --- | --- | --- | --- |
| **Exploring the health and illness experience** | | | | | |
| 1.The health worker greeted me in a way that made me feel comfortable | <5  0.00 | <5  1.00 | 9  3.00 | 118 39.33 | 170 56.67 |
| 2. The health worker used a language that I could understand | <5  0.00 | <5  0.00 | <5  0.00 | 50 16.67 | 250 83.33 |
| 3. The health worker encouraged me to express my thoughts concerning my health/illness | <5 0.67 | 10 3.33 | <5  1.00 | 203 67.67 | 82 27.33 |
| 4. I was able to discuss my reasons for coming today | <5 0.67 | <5  1.00 | <5  0.67 | 167 55.67 | 126 42.00 |
| 5. I discussed why it was important for me to come to the facility (i.e. and not stay at home or visit chemist…) | 14 4.67 | 164 54.67 | 38 12.67 | 74 24.67 | 10 3.33 |
| 6. The health worker listened carefully and was interested in what I thought the situation / problem was | <5 0.67 | 8  2.67 | 29 9.67 | 232 77.33 | 29 9.67 |
| 7. I was able to describe my symptoms | <5 0.67 | <5  1.00 | <5  0.67 | 174 58.00 | 119 39.67 |
| 8. The health worker listened carefully and was interested when I talked about my symptoms | <5 0.67 | <5  1.33 | 44 14.67 | 221 73.67 | 29 9.67 |
| 9. The health worker asked me what I believe is causing my medical symptoms | 7 2.33 | 138 46.00 | 6  2.00 | 127 42.33 | 22 7.33 |
| 10. The health worker was interested in what treatment I had before | <5 1.00 | 98 32.67 | <5  0.33 | 154 51.33 | 44 14.67 |
| 11. I was able to explain treatment that I had got before | <5 1.00 | 97 32.33 | <5  0.33 | 113 37.67 | 86 28.67 |
| 12. The health worker was able to look back at my health records and ask questions | 6 2.00 | 121 40.33 | <5  0.67 | 110 36.67 | 61 20.33 |
| 13. The health worker was interested in what I wanted to be done | <5 1.33 | 149 49.67 | 16 5.33 | 119 39.67 | 12 4.00 |
| 14. I answered all the health worker’s questions honestly | <5 0.67 | 32 10.67 | <5  1.00 | 173 57.67 | 90 30.00 |
| 15. The health worker understood what I had to say | <5 0.67 | 39 13.00 | 39 13.00 | 207 69.00 | 13 4.33 |
| 16. The health worker explained what the problem was | 12 4.00 | 125 41.67 | <5  1.00 | 132 44.00 | 28 9.33 |
| 17. The health worker explained what the cause(s) of the problem was (were) | 12 4.00 | 162 54.00 | <5  1.33 | 100 33.33 | 22 7.33 |
| **Understanding the whole person** | | | | | |
| 1. The health worker showed care and concern about me as a person | <5 0.00 | 15 5.00 | 22 7.33 | 250 83.33 | 13 4.33 |
| 2. The health worker was interested in the effect of the problem/condition on my family or personal life | 19 6.33 | 238 79.33 | 12 4.00 | 31 10.33 | <5  0.00 |
| 3. The health worker was interested in the effect of the problem/condition on everyday activities | 25 8.33 | 227 75.67 | 10 3.33 | 37 12.33 | <5  0.33 |
| 4. I could go to this health worker for help with a personal or emotional problem | 19 6.33 | 111 37.00 | 29 9.67 | 138 46.00 | <5  1.00 |
| 5. I’m confident that the health worker knows me and my history | 36 12.00 | 186 62.00 | 33 11.00 | 43 14.33 | <5  0.67 |
| 6. I’m confident that the health worker understands my emotional needs | 49 16.33 | 161 53.67 | 44 14.67 | 44 14.67 | <5  0.67 |
| 7. I’m confident that the health worker understands my cultural and spiritual preferences | 64 21.33 | 205 68.33 | 25 8.33 | 5  1.67 | <5  0.33 |
| 8. I’m confident that the health worker knows about my responsibilities at home, work or school | 67 22.33 | 188 62.67 | 23 7.67 | 22 7.33 | <5  0.00 |
| **Finding common ground / shared decision making** | | | | | |
| 1. The health worker encouraged me to ask questions | 9 3.00 | 190 63.33 | 11 3.67 | 85 28.33 | 5  1.67 |
| 2. I felt comfortable to ask questions | 7 2.33 | 177 59.00 | 17 5.67 | 95 31.67 | <5  1.33 |
| 3. The health worker responded to my questions | 5 1.67 | 178 59.33 | 18 6.00 | 96 32.00 | <5  1.00 |
| 4. The health worker and I discussed together what the problem was | 16 5.33 | 192 64.00 | 30 10.00 | 61 20.33 | <5  0.33 |
| 5. The health worker discussed treatment options with me | 20 6.67 | 247 82.33 | <5  0.67 | 28 9.33 | <5  1.00 |
| 6. I gave my opinion (agreement or disagreement) about the types of tests or treatment that my health worker ordered The health worker handled any doubts about the tests or treatment that were recommended | 26 8.67 | 232 77.33 | <5  1.00 | 39 13.00 | <5  0.00 |
| 7. We came up with the goals of treatment / health care plan | 16 5.33 | 217 72.33 | 27 9.00 | 39 13.00 | <5  0.33 |
| 8. The health worker checked to be sure I understood everything | 9 3.00 | 201 67.00 | 9  3.00 | 79 26.33 | <5  0.67 |
| 9. The health worker checked to be sure the treatment plan was manageable for me | 14 4.67 | 197 65.67 | 8  2.67 | 77 25.67 | <5  1.33 |
| 10. We discussed next steps, including any follow-up plans | 13 4.33 | 246 82.00 | 8  2.67 | 33 11.00 | <5  0.00 |
| 11. The health worker handled any doubts that I had | 17 5.67 | 163 54.33 | 35 11.67 | 85 28.33 | <5  0.00 |
| 12. We discussed our respective roles (the health worker and the patient) in my health care plan | 16 5.33 | 220 73.33 | 7  2.33 | 57 19.00 | <5  0.00 |
| 13. The health worker encouraged me to take the role I wanted in my own care | 14 4.67 | 196 65.33 | 13 4.33 | 77 25.67 | <5  0.00 |
| 14. The health worker involved me in decisions as much as I wanted | 12 4.00 | 197 65.67 | 30 10.00 | 59 19.67 | <5  0.67 |
| 15. The health worker gave me all the information I needed | <5 1.00 | 103 34.33 | 66 22.00 | 121 40.33 | 7  2.33 |
| **Prevention and health promotion** | | | | | |
| 1. The health worker talked about ways to lower the risk of future illness | 22 7.33 | 142 47.33 | <5  1.00 | 121 40.33 | 12 4.00 |
| 2. Th health worker advised me how to prevent future health problems (diet, health habits mentioned exercise, safe sex as relevant to illness or condition…) | 27 9.00 | 141 47.00 | <5  1.00 | 117 39.00 | 12 4.00 |
| **The relationship between the patient and the health worker** | | | | | |
| 1. The health worker was sympathetic | <5 0.33 | <5 0.00 | 9  3.00 | 184 61.33 | 106 35.33 |
| 2. The health worker was caring and compassionate | <5 0.00 | <5  1.67 | 30 10.00 | 256 85.33 | 9  3.00 |
| 3. I showed the health worker respect and accepted them as a person | <5 0.00 | <5  0.33 | <5  0.00 | 220 73.33 | 79 26.33 |
| 4. The health worker made me feel at ease | <5 0.00 | 5  1.67 | 33 11.00 | 243 81.00 | 19 6.33 |
| 5. The health worker respects me | <5 0.00 | <5  0.67 | 20 6.67 | 255 85.00 | 23 7.67 |
| 6. I trust the health worker to make good decisions about my health care | <5 0.00 | 15 5.00 | 19 6.33 | 249 83.00 | 17 5.67 |
| 7. This health worker cares more about the cost than what is needed for my health* | 127 42.33 | 143 47.67 | 16 5.33 | 14 4.67 | <5  0.00 |
| 8. This health worker would always tell me the truth about my health, even if it was bad news | 5 1.67 | 12 4.00 | 10 3.33 | 194 64.67 | 79 26.33 |
| 9. If a mistake was made in my treatment, my health worker would try to hide it from me* | 38 12.67 | 86 28.67 | 63 21.00 | 93 31.00 | 20 6.67 |
| 10. This health worker can take care of almost any medical problem I might have | 6 2.00 | 54 18.00 | 65 21.67 | 167 55.67 | 8  2.67 |
| 11. I feel this health worker treated me differently because of my ethnicity* | 111 37.00 | 174 58.00 | 12 4.00 | <5  1.00 | <5  0.00 |
| 12. I feel this health worker treated me differently because of my level of education* | 114 38.00 | 170 56.67 | 16 5.33 | <5  0.00 | <5  0.00 |
| 13. I feel this health worker treated me differently because of my ability to pay* | 122 40.67 | 161 53.67 | 16 5.33 | <5  0.33 | <5  0.00 |
| **Patient reported outcomes** | | | | | |
| **Patient satisfaction: Regarding your visit to the facility today, how do you feel about** | Very Satisfied | Somewhat satisfied | Neutral/ undecided | Unsatisfied | Very Unsatisfied |
| 1. The number of health workers | <5 0.00 | 17 5.67 | <5  0.67 | 197 65.67 | 84 28.00 |
| 2. How the health worker communicated with you | <5 0.00 | <5  0.33 | 6  2.00 | 117 39.00 | 176 58.67 |
| 3. Your treatment or health plan | 10 3.33 | 61 20.33 | 78 26.00 | 131 43.67 | 20 6.67 |
| 4. The advice given and follow up | 38 12.67 | 119 39.67 | 41 13.67 | 89 29.67 | 13 4.33 |
| 5. Would you come back to this facility | <5 1.33 | <5 0.00 | <5  0.00 | <5  0.00 | 296 98.67 |
| 6. Would you like to see the same health worker on your next visit | 5 1.67 | <5  0.00 | <5  0.00 | <5  0.00 | 295 98.33 |
| 7. Would you refer your friend or family to this facility | <5  1.33 | <5  0.00 | <5  0.00 | <5  0.00 | 296 98.67 |
| **Patient Enablement: As a result of your visit today, do you feel confident that you are:** | Very confident | Sort of confident | Undecided | Not so confident | Not at all confident |
| 1. Able to cope with life? | <5 0.00 | <5  0.67 | 60 20.00 | 220 73.33 | 18 6.00 |
| 2. Able to understand your illness? | <5 0.33 | <5  0.33 | 50 16.67 | 230 76.67 | 18 6.00 |
| 3. Able to cope with your illness? | <5 0.00 | <5  0.67 | 62 20.67 | 217 72.33 | 19 6.33 |
| 4. Able to keep yourself healthy? | <5  0.00 | <5  1.33 | 34 11.33 | 218 72.67 | 44 14.67 |
| 5. Confident about your health? | <5 0.33 | <5  0.67 | 36 12.00 | 218 72.67 | 43 14.33 |
| 6. Able to help yourself? | <5  0.00 | <5  0.00 | 38 12.67 | 230 76.67 | 32 10.67 |
| *These questions were negatively worded. They were asked as displayed on the table and responses reversed during analysis to stay uniform to the interpretation that positive responses meant better PCC scores | | | | | |
