# Supplementary material for: Patient perspectives on interpersonal aspects of healthcare and patient-centeredness at primary health facilities: A mixed methods study in rural Eastern Uganda
Source: PLoS One. 2020 Jul 30;15(7):e0236524. doi: 10.1371/journal.pone.0236524 (PMC7392339; doi:10.1371/journal.pone.0236524)
Supplement: Attachment — Submitted filename: Response to reviewers. PCC in Uganda.docx [file pone.0236524.s005.docx]

**Journal Requirements:**

**When submitting your revision, we need you to address these additional requirements.**

**1. Please ensure that your manuscript meets PLOS ONE's style requirements, including those for file naming. The PLOS ONE style templates can be found at**

[**https://journals.plos.org/plosone/s/file?id=wjVg/PLOSOne_formatting_sample_main_body.pdf**](https://journals.plos.org/plosone/s/file?id=wjVg/PLOSOne_formatting_sample_main_body.pdf) **and**

[**https://journals.plos.org/plosone/s/file?id=ba62/PLOSOne_formatting_sample_title_authors_affiliations.pdf**](https://journals.plos.org/plosone/s/file?id=ba62/PLOSOne_formatting_sample_title_authors_affiliations.pdf)

The format and file naming instructions have been updated

**2. We note that you have indicated that data from this study are available upon request. PLOS only allows data to be available upon request if there are legal or ethical restrictions on sharing data publicly. For information on unacceptable data access restrictions, please see** [**http://journals.plos.org/plosone/s/data-availability#loc-unacceptable-data-access-restrictions**](http://journals.plos.org/plosone/s/data-availability#loc-unacceptable-data-access-restrictions)**.**

**In your revised cover letter, please address the following prompts:**

**a) If there are ethical or legal restrictions on sharing a de-identified data set, please explain them in detail (e.g., data contain potentially identifying or sensitive patient information) and who has imposed them (e.g., an ethics committee). Please also provide contact information for a data access committee, ethics committee, or other institutional body to which data requests may be sent.**

**b) If there are no restrictions, please upload the minimal anonymized data set necessary to replicate your study findings as either Supporting Information files or to a stable, public repository and provide us with the relevant URLs, DOIs, or accession numbers. Please see** [**http://www.bmj.com/content/340/bmj.c181.long**](http://www.bmj.com/content/340/bmj.c181.long) **for guidelines on how to de-identify and prepare clinical data for publication. For a list of acceptable repositories, please see** [**http://journals.plos.org/plosone/s/data-availability#loc-recommended-repositories**](http://journals.plos.org/plosone/s/data-availability#loc-recommended-repositories)**.**

**We will update your Data Availability statement on your behalf to reflect the information you provide.**

The anonymized and de-identified quantitative patient exit data set is provided as a supplementary file and quotes used are provided in the text. Interview audio files and transcripts are governed under the Institute of Tropical Medicine (ITM) data sharing and open access policy. Access requests for ITM research data can be made to ITM’s central point for research data access by means of submitting a completed Data Access Request Form. These requests will be reviewed for approval by ITMs Data Access Committee, with further approval from the ITM Research Ethics *Committee. Please see this link for more information* [*https://www.itg.be/F/data-sharing-open-access*](https://www.itg.be/F/data-sharing-open-access)*. Contact information for ITM data access committee is* [*eb.gti@sseccaatadhcraeserMTI*](mailto:dev@null)*.*


**Review Comments to the Author**

**Reviewer #1: Well written paper on an important aspect of health care which I think should be accepted.**

Thank you for your gracious comments and for taking your time to read and review our manuscript

**I just have a couple of comments:
The result sub-sections (p17,21 and 28) would be improved by using the same titles as used in the introductory list on page 16.**

Thank you for this observation, the sub titles have now been edited to reflect the sub-topics in the introduction to the result section.

**Generally small numbers (i.e. less than 5) are not included in summary tables because of confidentiality and reliability. Suggest either collating the responses or replacing the small numbers with <5.**

We have made the decision to replace values less than 5 with <5 for content in the manuscript and supplementary file 3. However, we retain the values less than 5 for the patient exit interview data set (supplementary file 4) to enable replication of analysis.

**Need to consistently abbreviate patient centered care. Sometimes abbreviated and sometime provided in full.**

We have abbreviated PCC in the text but left it in full

- for the first mention in the abstract, first mention in the introduction,
- in the sub-titles, figure titles, table titles, supplementary file titles and,
- in instances where we are referring to patient centeredness or patient centered character where using the abbreviation may change the meaning.

**The discussion although generally relevant to the paper could be written more concisely to lead to the conclusions.**

We have reworked the discussion, also shortening it. We hope that this section is now a better read, and matches the conclusion.
**Reviewer #2: This is a mixed methods paper considering patient perspectives on interpersonal aspects of healthcare and patient-centredness in Uganda.

This is a well presented manuscript.**

Thank you for your kind words and comprehensive review.

**In the abstract I was less clear about the first sentence (line 30/31) – the results section in Abstract could be strengthened for impact and clarity.**

We have now re-written the results section to emphasize on the key findings of the study in the abstract, we also emphasise the structure of the results section of the abstract in line with the conceptual framework and result section of the manuscript.

**Some statements are made in the manuscript that need to be backed up by the literature e.g. line 57-58.**

We have re-worded the section to present how historically, quality improvement strategies and assessments of primary health care performance in LMICs have focused on health care providers, with little or no attention to consumer perspectives. We have also now provided the accompanying references.

**It would also be helpful to distinguish between the Countries of origin of studies. You nicely situate the Ugandan interventions.**

In the discussion, we have now made clear the countries of origin of studies in cases where it is one particular country, but for reviews, we have not mentioned all the countries considered in the reviews but we mention the regions e.g. sub-Saharan Africa.

**Characteristics of the healthcare facilities (Table 1) seems a little out of place in methods section. Can perhaps move to results or provide as a supplementary table from Introduction.**

Thank you for this suggestion, we have moved the characteristics of facilities to the first section of the results, therefore table 1 is now the summary of data collected and Table 2 the characteristics of facilities, we hope this improves the transition from the methods to the results section.

**Really nice Figure 1 and Figure 2.**

Thank you we appreciate you recognising our efforts

**You do not highlight male respondent numbers as a limitation. For this paper it may be better to focus only on the female respondents and orient the paper findings wholly to female aspects. If kept in, please discuss how respondent numbers of males might be so low/why.**

We believe it would be disadvantageous not to include male patients and care givers because it would go against our random selection of patients. We hypothesise that the respondent numbers of males is low because in primary health care facilities in LMICs most care seekers and care givers would be female. Furthermore, the higher number of female interviewees may also be because of our sampling of patients seeking maternal and child health who would - as expected - be predominantly female. We included this in the limitations section, with the appropriate explanation.

**Reference 2 and 21 – please check spelling of WHO name (Organization)**

This has now been corrected

**Data underlying the findings is not provided, some restrictions apply. A link is provided to the source of the data.**

The anonymised and de-identified quantitative patient exit data set is now provided as supplementary file 4

**[NOTE: If reviewer comments were submitted as an attachment file, they will be attached to this email and accessible via the submission site. Please log into your account, locate the manuscript record, and check for the action link "View Attachments". If this link does not appear, there are no attachment files.]

While revising your submission, please upload your figure files to the Preflight Analysis and Conversion Engine (PACE) digital diagnostic tool,** [**https://pacev2.apexcovantage.com/**](https://pacev2.apexcovantage.com/)**. PACE helps ensure that figures meet PLOS requirements. To use PACE, you must first register as a user. Registration is free. Then, login and navigate to the UPLOAD tab, where you will find detailed instructions on how to use the tool. If you encounter any issues or have any questions when using PACE, please email PLOS at** [**figures@plos.org**](mailto:figures@plos.org)**. Please note that Supporting Information files do not need this step.**

**When you're ready to submit your revision, log on to** [**https://www.editorialmanager.com/pone/**](https://www.editorialmanager.com/pone/) **and select the 'Submissions Needing Revision' folder to locate your manuscript file.**
